# Extracellular vesicles in liquid biopsies: there is hope for oral squamous cell carcinoma

**DOI:** 10.20517/evcna.2024.29

**Published:** 2024-12-07

**Authors:** Leanne Lee Leung, Xinyu Qu, Bojie Chen, Jason YK. Chan

**Affiliations:** Department of Otorhinolaryngology, Head and Neck Surgery, The Chinese University of Hong Kong, Hong Kong Special Administrative Region, Hong Kong 00000, China.

**Keywords:** Oral cancer, liquid biopsy, saliva, plasma/serum, lymphatic fluid, EVs, extracellular vesicles

## Abstract

Current approaches to oral cancer diagnosis primarily involve physical examination, tissue biopsy, and advanced computer-aided imaging techniques. However, despite these advances, patient survival rates have not significantly improved. Hence, there is a critical need to develop minimally invasive tools with high sensitivity and specificity to improve patient survival and quality of life. Liquid biopsy is a non-invasive, real-time method for predicting cancer status and potentially serves as a biomarker source for treatment response. Liquid biopsy includes rich biologically relevant components, such as circulating tumor cells, circulating tumor DNA, and extracellular vesicles (EVs). EVs are particularly intriguing due to their relatively high abundance in most biofluids, with the potential to identify specific cargo derived from circulating tumor EVs. Moreover, normal cells in lymph nodes can uptake EVs, fostering a pre-metastatic microenvironment that facilitates lymph node metastases - a common occurrence in oral cancers.

This review encompasses English language publications over the last twenty years, focusing on methods for isolating EVs from saliva, blood, and lymphatic fluids, as well as the collection methods employed. Seventeen cases met the inclusion criteria according to ISEV guidelines, including 10 saliva cases, 6 blood cases, and 1 lymphatic fluid case. This review also highlighted research gaps in oral squamous cell carcinoma (OSCC) EVs, including a lack of multi-omics studies and the exploration of potential EV markers for drug resistance, as well as a notable underutilization of microfluidic technologies to translate liquid biopsy EV findings into clinical applications.

## INTRODUCTION

According to the Globocan Cancer Observatory, the incidence of oral cancer, as part of head and neck cancers/squamous cell carcinomas (HNSCC), has been rising annually. Among these cases, Asia accounts for the highest incidence and mortality rates, followed by Europe, North America, Latin America and the Caribbean, and Africa^[1]^. Males are at two-fold higher risk than females for developing oral squamous cell carcinoma (OSCC). OSCC is the dominant subtype of oral malignancy globally. The anatomical subsites of OSCC are diverse, including the buccal mucosa, tongue, floor of the mouth, gums, hard palate, lips, and retromolar trigone^[2]^. The pathogenesis of OSCC is complicated, involving multiple molecular mechanisms, gene mutations, and altered levels of proteins and metabolites^[3]^. There are a variety of risk factors that lead to OSCC, such as consumption of tobacco, alcohol, and betel nut chewing^[4]^. Despite optimizing treatments with surgery, radiotherapy and/ or chemotherapy, the survival rate remains at 40%. Therefore, there is an urgent need for methods to identify early OSCC cases to improve outcomes^[5]^.

The current gold standard for OSCC diagnosis is histopathological assessment. However, histopathology has limitations, as it cannot monitor the dynamic tumor response to therapeutic treatments and relapse from drug resistance^[6]^. Liquid biopsies are preferable due to their quick and non-invasive collection, making sample acquisition and storage simple^[7]^. Moreover, liquid biopsies enable the detection of tumor-related markers for identifying individuals at risk for OSCC, as well as for monitoring patient prognosis and response to treatments^[7,8]^.

Oral biopsy collection has been used to diagnose a wide range of conditions and diseases. For example, a recent report described 47 studies that revealed the diagnostic potential of salivary/crevicular fluid EVs, especially in head and neck and oral cavity diseases. These include conditions such as gingivitis, oral lichen planus, oral squamous cell carcinoma, oropharyngeal cancer detection, orthodontic root resorption, periodontitis, peri-implantitis, Sjögren syndrome, and various systemic diseases^[9]^.

**Figure 1:**
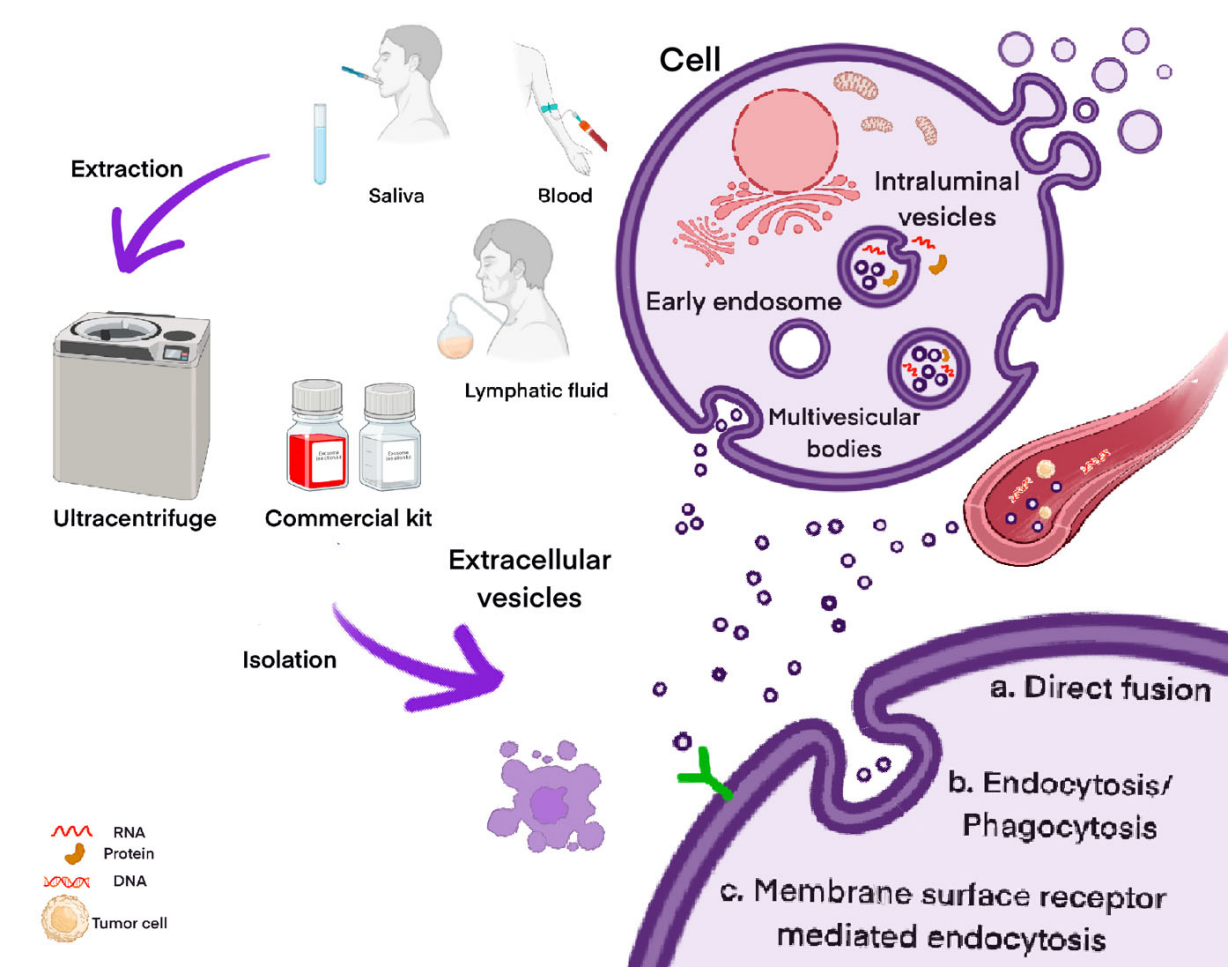
EVs extracted from different types of liquid biopsies^[15-18]^. EVs: extracellular vesicles.

Various biofluids can be used for liquid biopsies, such as cerebrospinal, peritoneal, pleural, and lymphatic fluids, as well as blood, saliva, mucosa, and urine^[10]^. Among these, the detection of EVs in liquid biopsies has garnered significant interest in solid tumors over the last decade. EVs as a class of circulating particles, consist of apoptotic bodies, microvesicles, and exosomes^[11]^. Exosomes are classified as small EVs ranging from 40-200 nm in diameter, containing mRNA, microRNA, lipids, and proteins. These nanosized particles are secreted from all cells and are taken up by both nearby and distant organ receptor cells via surface proteins or other endocytotic mechanisms^[10,12,13]^. The release of EVs in liquid biopsies facilitates the detection of various genetic materials within tumor cells. Unlike circulating DNA, EVs are enclosed by a lipid bilayer, which aids in preserving the quality of EV content, particularly nucleic acids^[14]^. Advancements in understanding the clinical applications of liquid biopsies indicate that EVs, serving as OSCC biomarkers, offer promising prospects for early diagnosis [Figure 1]. In general, OSCC-derived EVs can be collected from saliva, blood, and lymphatic fluids by various centrifugations or chemical precipitations.

Numerous publications have explored the diagnostic potential of EVs in the early detection of complex oral diseases such as OSCC, periodontitis, gingivitis, and oral lichen planus, as previously mentioned. In addition, the therapeutic potential of EVs has been widely reported, including their roles in angiogenesis, bone regeneration, cancer therapy, cementoblast regeneration, endo/pulp generation, immune cell behavior, nerve regeneration, oral and periodontal pathogens, orthodontic tooth movement/resorption, and periodontal regeneration^[9]^.

Although many studies have demonstrated the potential of saliva and blood EV markers in detecting OSCC, there are no set guidelines on the extraction of purified EVs for accurate marker detection. For obtaining saliva, there is no standardized protocol for collection, whether by swab, oral rinse, or direct unstimulated saliva. However, there is a general consensus for the use of protease inhibitors or dilution with PBS during different centrifugation processes to preserve saliva EVs. Furthermore, there are no standardized methods for detecting potential EV markers. Most studies detect marker expression through the measurement of gene expression (qPCR), DNA (microarray) or proteomic analysis (ELISA). Purification processes for blood EVs have been extensively studied in comparison to those of saliva EVs, as reports show that size exclusion chromatography provides optimal stability for EV isolation. However, there are no guidelines on which potential biomarkers are suitable for clinical detection. Additionally, there is a lack of studies investigating whether EV biomarker expression varies across different stages of OSCC.

**Figure 2:**
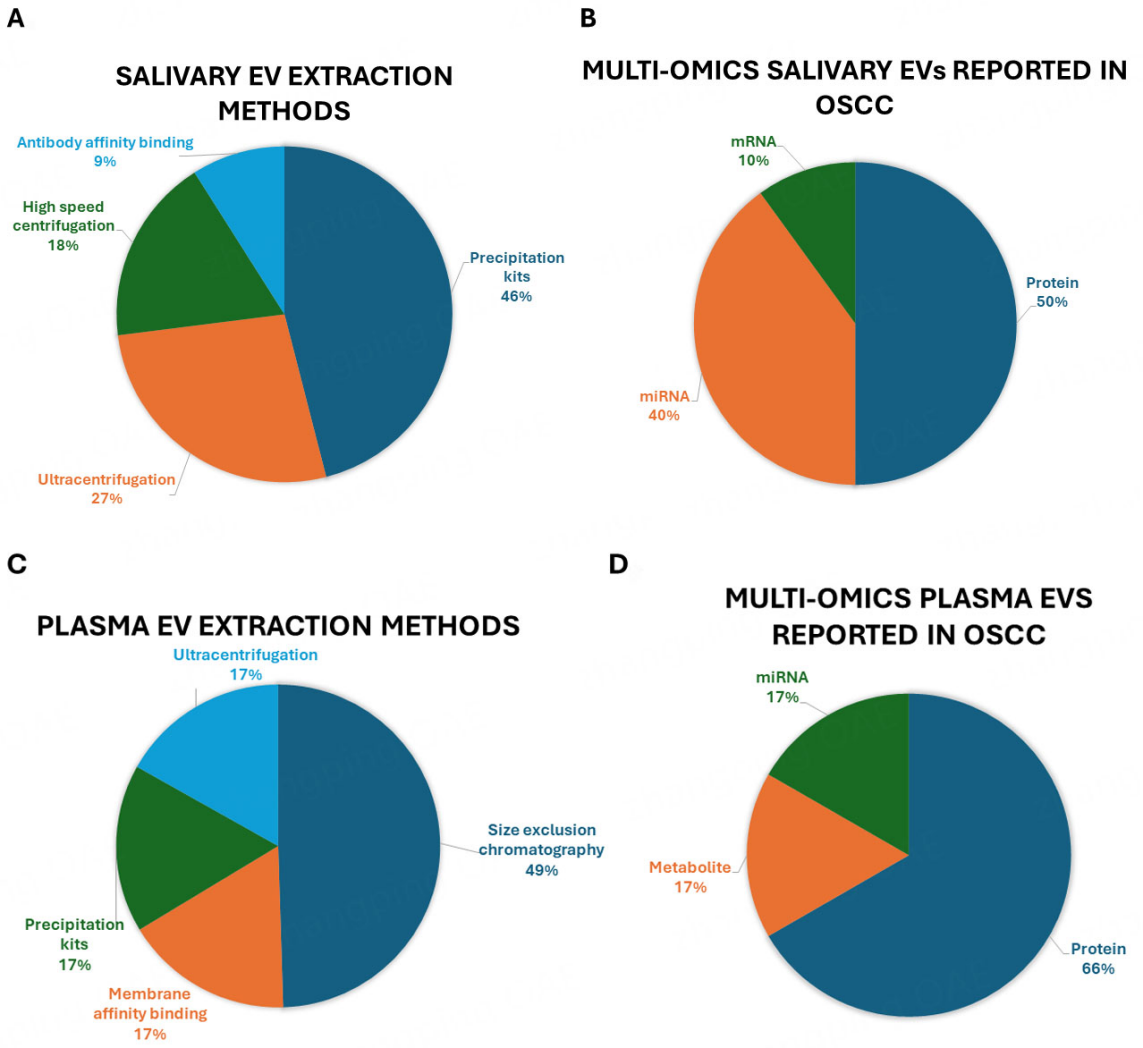
Illustration of summarized potential EV biomarker studies in salivary and plasma samples. A: Salivary EV extraction methods; B: Multi-omics salivary EVs reported in OSCC; C: Plasma EV extraction methods; D: Multi-omics plasma EVs reported in OSCC. EVs: extracellular vesicles; OSCC: oral squamous cell carcinoma.

**Figure 3:**
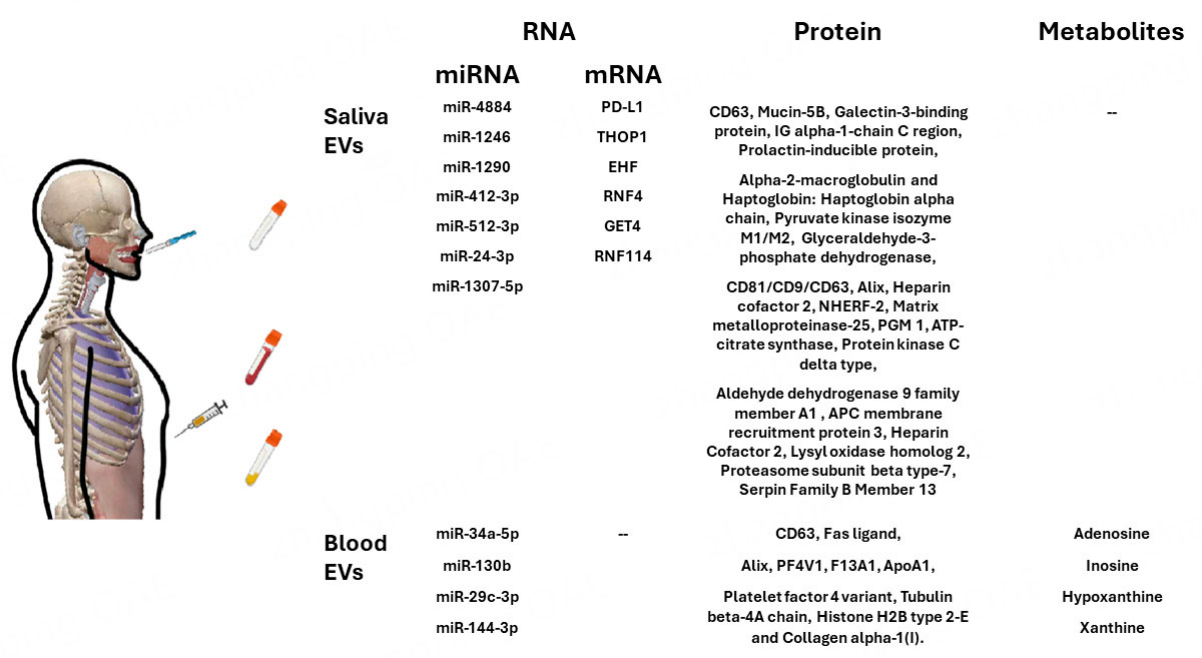
Potential EV biomarkers identified in OSCC studies, derived from saliva and blood samples. EVs: extracellular vesicles; OSCC: oral squamous cell carcinoma.

In this review, we first summarize the studies over the last two decades on how to isolate EVs from biofluids from OSCC patients according to MISEV2023 guidelines [Figure 2]. We focus on discussing methods and detection efficiency. In addition, we summarize the EV nucleic acids extracted from liquid biopsies and discuss the profiling of OSCC samples [Figure 3]^[15-17,19-30]^. Finally, we discuss the current status of EV research, identify existing gaps, and explore potential advancements in future EV detection.

## CURRENT METHODS TO ISOLATE TUMOR-DERIVED EXTRACELLULAR VESICLES FROM BIOFLUIDS

At present, many strategies and technologies exist for isolating tumor-derived EVs from biofluids, such as standard methods of ultracentrifugation, gradient density ultracentrifugation, ultrafiltration, tangential flow filtration, and size exclusion chromatography. Recently, microfluidic chip-based sorting techniques have also been introduced^[31]^. In this article, we aim to discuss the pros and cons of these EV separation methods.

### Ultracentrifugation

To date, ultracentrifugation remains the most common method to isolate EVs. The approach relies on separating particles by size and density from other proteins, large vesicles, and putative impurities^[32]^. The process involves high-speed centrifugation (> 100,000 x *g*) over extended durations, typically 70 min per spin, followed by EV pellet washing to separate crude particles of similar density. However, the size and shape of EVs must be validated by other applications. Despite its widespread use due to its applicability to most biofluids and overall affordability, ultracentrifugation is accompanied by several drawbacks. Ultracentrifugation can lead to co-precipitation of protein aggregates and other subcellular organelles^[33,34]^, impacting the quality of subsequent analytical techniques such as mass spectrometry^[35]^. Moreover, this technique can compromise EV membrane integrity and thus impact biological activity. Hence, this presents challenges for large-scale production due to its low yield and purity^[35]^.

To enhance purification and remove impurities, density gradient ultracentrifugation was developed, which combines ultracentrifugation with the use of 30% sucrose or iodixanol to create distinct density layers. EVs are isolated where EV density equals the gradient density in the centrifugal field gradients, at a range of 20%-30%. This method aids in the separation of protein contaminants, thus improving yield and preserving biological properties ^[36,37]^. However, density gradient ultracentrifugation requires advanced technical skills for cushion operations. Additionally, long centrifugation times of up to 16 h are required to separate EVs into gradient fractions, which can hinder clinical efficiency^[37,38]^ .

### Ultrafiltration

Ultrafiltration separates EVs based on molecular size, i.e., diameter, and molecular weight, i.e., pore size, as membrane filters with specific pore sizes remove impurities, allowing for the collection of purified EVs. EVs are fractionated from larger or smaller entities by selection of pore sizes that either allow EV passage through the membrane, or the EVs are retained if the pore size is smaller than the EVs. The ultrafiltration method offers advantages such as ease of use and relatively short processing times (0.5-1 h), but may have volume limitations depending on the filtration device. Ultrafiltration does not require specific equipment and reagents for EV collection. Studies have shown that membrane filters with a 10kDa cutoff size yield the highest recovery rates of EVs^[39-41]^, but the retained materials could be anything larger than small (10kDa) proteins or nucleic acids.

Currently, dead-end filtration and tangential flow filtration are commonly used techniques for isolation. Dead-end filtration involves a one-way flow where the liquid permeates through the filter membrane. However, a drawback is that the filter membrane can easily become blocked by large impurities or even EV conglomerates, limiting its application to small-scale EV production^[42]^. Tangential flow filtration is employed for large-scale production, where a liquid reagent permeates a membrane filter, allowing the separation of particles from a colloidal matrix. The liquid flows parallel to the membrane surface, as opposed to dead-end filtration^[43]^. However, one drawback is that impurities can clog the membrane pores, thereby reducing the yield of EVs.

### Size exclusion chromatography

Recently, more studies have reported using size exclusion chromatography (SEC) to fractionate EVs from complex fluids such as liquid biopsies. SEC columns contain porous beads that facilitate the separation of plasma protein contamination from EVs, with the elution of molecules occurring in a size- and time-dependent manner^[44-46]^. Molecules larger than the pore size elute first due to their inability to enter the pores, whereas smaller molecules can penetrate the pores and, therefore, elute later. Due to the gentle, gravity-driven nature of this method, SEC preserves vesicle structure and biological properties. In addition, SEC allows for the purification of EVs from small sample volumes, ranging from 500 µL to 2 mL^[47]^.

Compared to traditional EV isolation methods, SEC elutes what are described as pure EVs quickly and simply. However, it has certain limitations: all EV fractions are required to be collected, the EV size needs to be manually determined^[47]^, and it is a diluting method where EVs may be distributed across multiple fractions. The lower yield of eluted EVs affects the concentration of EV-bound proteins and genetic materials, such as miRNAs^[48]^. Additionally, the beads cannot differentiate between similarly sized EVs, especially exosomes and microvesicles^[49]^. Despite this method’s drawbacks, SEC efficiently elutes relatively pure EVs while preserving their function and integrity; hence, this method is recognized in Minimal Information for Studies of Extracellular Vesicles 2023 (MISEV 2023) for the development of biomarker discovery applications.

### Microfluidics

Microfluidics-based methods are rising as innovative techniques that combine microscale channels (on chips) with size, density and/or immunoaffinity capture to isolate EVs^[50]^. A key advantage of microfluidics is that it requires very small volumes of biofluids to extract EVs, hence fast purification and high-purity EV capture, facilitating high-throughput analysis^[51]^, which requires sensitive detection and readout capabilities. However, the drawbacks include the high cost of devices and the need for specialized equipment^[52]^. Although microfluidic technologies are not yet considered standard for EV isolation, their development shows promising potential^[53]^.

In this review, we include studies involving OSCC patient samples that employ recognized EV isolation according to MISEV2023^[54]^. Although MISEV2023 does not list specific guidelines for liquid biopsies, some ISEV position publications have listed the suggested standards for sample validation^[14,32,55]^. The following isolation and characterization methods are noted and included in this review article.

### Classical techniques

EVs in biofluid samples may be separated using size- and/or density-based methods either alone or in combination with each other. These methods include ultracentrifugation, density gradient (iodixanol or sucrose) ultracentrifugation, SEC, and ultrafiltration. Biomarker-specific EV capture methods such as immunoaffinity binding, usually attached to beads or other immobilized structures, are also effective.

### Precipitation

Precipitation kits are recommended for faster and higher yields of EV separation. The matrix in these kits primarily consists of bound beads, antibodies, and polymers. However, the purity of EVs from matrix-precipitated samples is often lower due to the co-isolation of contaminant proteins that adhere to EVs^[33]^. EVs bound with matrix components might affect their effectiveness in downstream applications. EVs precipitated using these matrices have been observed to affect imaging analyses, exhibiting dark backgrounds and rough surfaces, resulting in low-contrast images^[34]^.

### Quantification methods

This article presents at least two different complementary techniques for the quantification of OSCC-derived EVs:

EV Composition and Visualization: The composition of isolated EVs can be complex. To visualize the size and structure of EVs, various forms of electron microscopy (EM) can be used, including scanning EM (SEM), transmission EM (TEM), and cryo-TEM. Scanning probe microscopy (SPM) also offers alternatives such as atomic force microscopy (AFM) and super-resolution microscopy^[35,36,56]^.

EV Quantification: To quantify EVs, nanoparticle tracking analysis (NTA) is one of the techniques suggested to determine size and concentration.

EV characterization: Three positive protein markers of EVs are reported in the review, including transmembrane proteins (tetraspanins CD9, CD63 and/or CD81), one cytosolic protein (TSG101, Flotillin-1 or -2, and HSP70), and one component of the corona protein [albumin, apolipoproteins-A1/2 (APOA1/2) and -B (APOB)]. Additionally, multi-angle light scattering and fluorescence detection bead flow cytometry. Next, bead-based flow cytometry is not truly quantitative unless one can determine the number of EVs bound to beads. Nanoflow cytometry can be quantitative but requires specialized equipment, adequate training, and instrument calibration^[37,38]^.

Regarding the common standard methods for collecting biofluid EVs, the following sections discuss various methods of collecting saliva and blood EVs, each of which can impact the quality of biomarkers. Additionally, the collection process and sample storage temperatures are summarized.

### Saliva EVs

Saliva, an acidic biological fluid, consists predominantly of water and various compounds such as enzymes, electrolytes, microbes, and mucus. It is secreted by major and minor salivary glands distributed throughout the oral cavity submucosa. Salivary glands, densely vascularized with a network of capillaries, facilitate substantial molecule exchange with blood circulation through passive and active cellular transporters and channels^[57]^. Saliva offers direct monitoring of the oral microenvironment and has been implicated in many oral inflammatory diseases, including gingivitis, periodontitis, and OSCC^[58-60]^.

Currently, standardized methods for collecting and analyzing saliva EVs are lacking. In the following section, we highlight studies that have isolated salivary EVs for potential biomarkers in OSCC. Furthermore, we discuss the technical challenges and future prospects of using saliva EVs as biomarkers in conventional OSCC diagnosis.

#### Salivary EVs as potential biomarkers in OSCC

Of the current literature, there are ten impactful salivary EV studies on potential biomarkers in OSCC. For EV isolation, most of the studies (40%) used precipitation reagents and kits such as ExoQuick-TC^TM^ (System Bioscience, Mountain View, CA, United States ) and Invitrogen^TM^ Total Exosome Isolation Reagent (ThermoFisher Scientific, Waltham, MA, USA). This was followed by ultracentrifugation (30%), high-speed centrifugation (20%), and/or antibody affinity binding (10%). Of the ten studies, the majority reported proteins as potential saliva EV biomarkers for OSCC (50%), followed by microRNAs (40%), and mRNAs (10%).

Precipitation-based isolation of EVs from saliva is commonly employed by researchers due to its simple process. This technique will be introduced in the following paragraphs. One of the studies on salivary EVs was reported in oral lichen planus (OLP), an oral disease with high potential malignancy. This disease manifests in the oral mucosa with white papules and has six typical patterns: atrophic, bullous, erosive, papular, plaque, and reticular forms^[61,62]^. Byun *et al.* used unstimulated whole saliva samples (5-10 mL) used for EV extraction by precipitation from 16 OLP patients and eight healthy controls^[15]^. The EVs were subsequently characterized by NTA, demonstrating round vesicle structures sized between 20-100 nm. Surface protein analysis by flow cytometry identified the presence of a characteristic EV marker, CD63. Additionally, miRNA microarray revealed 57 miRNAs exhibiting more than a two-fold difference between healthy individuals and OLP patients. Among the 57 miRNAs, miR-4884, miR-1246, and miR-1290 were further validated by qRT-PCR. Notably, miR-4884 was found to be increased (up to 98-fold) in OLP patient samples compared to healthy individuals^[15]^.

Salivary EVs may also play a crucial role in the early diagnosis of periodontitis, a condition associated with an increased risk of OSCC^[63,64]^. In a study by Yu *et al.*, unstimulated whole saliva samples (3-5 mL) were collected from 61 periodontitis patients and 30 healthy individuals. EV PD-L1 mRNA was quantified through qPCR, revealing that 74% of periodontitis patients had elevated PD-L1 mRNA expression^[16]^. Gai *et al.* collected salivary EVs from 21 OSCC patients and 11 non-cancer cases. Their findings indicated significantly higher levels of miR-412-3p and miR-512-3p in OSCC patients compared to those without cancer^[19]^. Similarly, He *et al.* isolated EVs from the saliva of 49 OSCC patients and 14 healthy individuals. Microarray analysis demonstrated a significant increase in the expression of miR-24-3p in EVs derived from OSCC patients compared to healthy individuals, which was further validated by quantitative real-time PCR^[20]^. In another study, Patel *et al.* using precipitation kits, isolated salivary EVs from eight OSCC patients and eight healthy individuals. Small RNA sequencing analysis revealed enhanced expression of miRNA-1307-5p in OSCC patient salivary EVs, consistent with elevated miRNA-1307-5p observed in patient-derived tumor tissues. High levels of miRNA-1307-5p were clinically related to tumor aggressiveness, chemoresistance, disease progression, and poor patient survival. Profiling of miRNAs and mRNAs identified that five mRNA targets were being suppressed (THOP1, EHF, RNF4, GET4, and RNF114) in agreement with high-affinity binding predictions for miRNA-1307-5p^[21]^.

Ultracentrifugation was commonly used for extracting salivary EVs. To explore the proteomic profiles of salivary EVs, Winck *et al.* studied 10 healthy individuals and isolated 24 OSCC salivary EV samples. Mass spectrometry analysis was performed to compare proteins detected in salivary EVs to those in whole saliva. Proteomic analysis revealed that OSCC patients had higher expression of eight proteins in salivary EVs compared to healthy individuals. The eight proteins (MUC-5B, Gal-3BP, IGHA1, PIP, α2M, Hp, PKM1/M2, and GAPDH) play important roles in immune responses in inflammation and chemotaxis. These findings suggest that salivary EVs may play a crucial role in chemotactic mechanisms and in attracting inflammatory cells^[22]^.

A study by Zlotogorski-Hurvitz detected morphological and molecular differences in salivary EVs between 36 OSCC patients and 25 healthy individuals. They showed that saliva from OSCC patients had a significantly higher EV concentration and a larger modal EV size compared to those from healthy individuals. Three-dimensional atomic force microscopy images of EV pellets highlighted a size difference between OSCC patient-derived EVs and those from healthy individuals. ELISA and Western blotting showed a differential abundance of EV markers, with lower expression of CD81 and CD9 and higher expression of CD63 in OSCC EVs compared to healthy EVs^[23]^. These findings have led subsequent OSCC studies to adopt similar validation methods for detecting EVs and related EV markers^[26]^.

Human Alix transcripts have been detected in human cancer tissues, but their expression in OSCC EVs was previously unknown. Nakamichi *et al.* analyzed salivary and serum EVs from 23 OSCC patients and compared them to 20 healthy controls, isolating the EV samples by ultracentrifugation. They found that salivary Alix protein levels in salivary EVs were significantly higher in OSCC patients compared to healthy controls^[24]^.

Sun *et al.* (2022) recently introduced a novel bifunctional magnetic bead technology with beads functionalized with titanium IV ions and 1,2-distearoyl-3-sn-glycerophosphoethanolamine (DSPE), a lipid analog. The beads offered high binding affinity, increased purity, and reduced processing time. The authors assessed the proteomic and phospho-proteomic profiles of 30 healthy individuals and 30 OSCC patients, identifying 2500 EV proteins and 1000 EV phosphoproteins. The authors subsequently analyzed EV proteins using high-speed sequencing called parallel accumulation-serial fragmentation, before and after surgical resection in patients with OSCC, with significant alterations observed in proteins such as heparin cofactor II (HCII), NHERF-2, matrix metalloproteinase-25 (MMP-25), PGM 1, ATP citrate lyase (ACLY), and protein kinase C-delta (PKCδ)^[25]^.

Salivary EVs have been proposed as potential early biomarker sources for OSCC. Bozyk *et al.* conducted a study involving three cohorts: cancer-free healthy controls (*n*=20), early-stage OSCC patients (*n*=10), and patients with oral potentially malignant disorders (OPMD) (*n*=20). Analysis of the samples using NTA with downstream mass spectrometry proteomics revealed that whole-mouth saliva collection yielded a higher concentration of EVs, compared to oral rinse EVs. Additionally, the study identified six significantly dysregulated proteins between OSCC and OPMD participants: aldehyde dehydrogenase 9 family member A1 (ALDH9A1), APC membrane recruitment protein 3 (AMER3), heparin cofactor II (HCII)), lysyl oxidase homolog 2 (LOXL2), proteasome subunit beta type-7 (PSB7), and serpin family B member 13 (SERPINB13). These proteins are related to cancer development and progression^[26]^. Tables 1 and 2 summarize the pretreatment of salivary EVs and their inclusion in OSCC, respectively, detailing the sample processing methods, EV isolation methods, detection methods, and potential biomarkers in EVs.

**Table 1:** Summary of salivary EVs pretreatment in OSCC

**Type of collection tube**	**Volume of sample**		**Sample filtering**	**Sample processing**	**Sample volume and temperature for storage after pretreatment**	**Reference**
50 mL conical tube		5-10 mL	No	500 µL sample mixed with precipitation solution in a 1:1 ratio, incubated overnight at 4 ℃ Samples were centrifugated at 1,500 x *g* for 15 min at 4 ℃	1 mL /--	Byun *et al.,* 2015^[15]^
50 mL Falcon tube		--	0.2 µm filter	Samples were diluted in a 1:1 ratio with PBS, and centrifuged at 3,000 x *g* for 15 min at room temperature to remove cells, debris, and bacteria Precipitation solution, 65 µL per 250 µL saliva, was mixed with the sample and incubated overnight The following day, samples were centrifuged at 3,000 x *g* for 30 min to precipitate EVs	--/ -80 ℃	Gai *et al.,* 2018^[19]^
1.5-mL tube aliquots		3-5 mL	No	Samples were kept on ice for no more than 60 min, and subsequently, centrifuged at 3,000 x *g* for 15 min EVs supernatant was mixed with precipitation solution at a 63:250 ratio and then refrigerated at 4 ℃ overnight for precipitation The mixture was then centrifuged twice at 1,500 x *g* for 30 min and 5 min, respectively, to remove the supernatant	--/ -80 ℃	Yu *et al.,* 2019^[16]^
Chilled 50-mL conical tubes		5 mL	No	Samples were immediately centrifugated at 2,600 x *g* for 30 min at 4 ℃ to remove cell debris, bacteria, and any food residuals 0.5-1 mL saliva sample was incubated with EV precipitation solution overnight at 4 ℃ The next day, samples were centrifugated at 1,500 x *g* for 30 min at 4 ℃. Pellets were recovered	--/ -80 ℃	He *et al.,* 2020^[20]^
Sterile tubes		--	No	Samples were centrifuged at 2,000 x *g* for 10 min at room temperature to remove cells and debris Samples were then diluted with PBS in a 1:1 ratio, and EVs were precipitated using a precipitation solution	--/ -80 ℃	Patel *et* *al.,* 2022^[21]^
1.5-mL tube aliquots		--	No	1 mL saliva was diluted 1:1 with PBS containing protease inhibitors and 1 mM DTT. Samples were then centrifuged sequentially at 200 x g for 5 min, 2,000 x *g* for 10 min, 3,500 x *g* for 10 min, and finally at 10,000 x *g* for 90 min at 4 ℃ to obtain the pellet	--/ -80 ℃	Winck *et* *al.,* 2015^[22]^
Sterile tubes		2-7 mL from cancer patients 5-20 mL from healthy controls	0.22- µm syringe filter	Samples were immediately centrifuged at 3,000 x *g* for 20 min at 4 ℃ to remove cells and debris Pooled samples were centrifuged at 12,000 x *g* for 20 min to remove residual organelles and cell fragments Next, 0.5 mL supernatant was diluted with PBS in a 1:1 ratio Samples were ultracentrifuged at 120,000 x *g* for 90 min at 4 ℃ twice to obtain the EV pellets	147 mL for oral cancer patients; 230 mL /-70 ℃	Zlotogorski- Hurvitz *et al.,* 2016^[23]^
--		1 mL	No	1 mL of sample was diluted 1:2 with PBS and centrifuged at 10,000 x *g* for 10 min at 4 ℃ Subsequently, samples were centrifuged at 100,000 x *g* for 70 min twice to obtain the EV pellet	--/--	Nakamichi *et al.,* 2021^[24]^
--		--	No	Samples were centrifuged at 2,500 x *g* for 15 min to remove cell debris, apoptotic bodies, and large aggregates. Saliva was thawed at 37℃, mixed with buffers, and then incubated with BiMBs beads for 1 h 200 µL of TEA buffer was added to elute EVs	Freeze dry	Sun *et al.,* 2023^[25]^
50 mL Falcon tube		300-400 μL	No	Samples were centrifuged at different speeds: 2,000 x *g* for 10 min to pellet cells and apoptotic bodies, 16,000 x *g* for 20 min to remove larger EVs, and finally, 120,000 x *g* for 3 hr to pellet EVs	50 μL /80 ◦C	Bozyk *et* *al.* 2023^[26]^

EVs: extracellular vesicles; OSCC: oral squamous cell carcinoma.

**Table 2:** Salivary EVs included in OSCC

**Isolation Method(s)** **U** **P** **O**	**Validation**	**EV Marker(s)**	**Detection method(s)**	**Potential biomarkers** **in EVs**	**Reference**
P	^a^Flow cytometry	CD63	miRNA Microarray	miR-4884	Byun *et al.,* 2015^[15]^
P	^a^^,^^b^Western Blotting	CD9, CD63, Alix, TSG101	^e^	miR-412-3p, miR-512-3p	Gai *et al.,* 2018^[19]^
P	^a^^,^^b^Western Blotting	CD9, CD81, CD63, Alix, TSG101	^e^	PD-L1 mRNA	Yu *et al.* 2019^[16]^
P	^a^^,^^b^Western Blotting	CD63, CD81, TSG101	Microarray, ^e^	miR-24-3p	He *et al.* 2020^[20]^
P	^a^^,^^b^Flow cytometry	CD9, CD81, CD63, CD47	Small RNA sequencing	miRNA-1307-5p	He *et al.,* 2022^[20]^
U	^a^^,^^b^Western Blotting	Flotillin-1	Shotgun proteomics analysis	Mucin 5B, Galectin-3-binding protein Ig alpha-1 chain C region, Prolactin-inducible protein, Alpha-2-macroglobulin Haptoglobin (Haptoglobin alpha chain), Pyruvate kinase isozyme M1/M2, Glyceraldehyde-3-phosphate dehydrogenase	Winck *et* *al.,* 2015^[22]^
U	^a^^,^^b^^,^^c^Western Blotting	CD9, CD81, CD63	^d^	CD63, CD9, CD81	Zlotogorski-Hurvitz *et al.,* 2016^[23]^
U	^a ^^,^^a^with immunogold labeling, ^b^Western Blotting,	CD9, CD81, CD63,	^d^	Alix	Winck *et* *al.,* 2021^[22]^
U, O^g^	^a^^,^^b^Western Blotting	CD9, CD81, TSG101	Proteomic analysis, Phosphopeptide enrichment	Hep2, MMP25, ACLY, KPCD	Sun *et al.,* 2023^[25]^
U	^a^^,^^b^Western Blotting	CD9, CD63, CD81,	Nano-^f^system	AL9A1, AMER3, HEP2, LOXL2, PSB7, SPB13	Bozyk *et* *al.,* 2023^[26]^

EVs: extracellular vesicles; OSCC: oral squamous cell carcinoma; U: ultracentrifugation; P: precipitation; O: others; ^a^TEM: transmission electron microscopy; ^b^NTA: nanoparticle tracking analysis; ^c^AFM: atomic force microscopy; ^d^ELISA: enzyme-linked immunosorbent assay; ^e^qPCR: quantitative polymerase chain reaction; ^f^LC-MS/MS: liquid chromatography-tandem mass spectrometry.

### Blood EVs

Blood samples serve as crucial liquid biopsy specimens for cancer diagnosis. Compared to traditional tissue extraction methods, blood sampling entails lower risks and facilitates faster detection of cancer at early stages and during recurrence^[65]^. Blood contains various sources of genetic material, including cell-free DNA, circulating tumor cells, and EVs. Current research emphasizes EVs from blood as biomarkers for cancer metastases, instead of cell-free DNA and circulating tumor cells, due to the abundance and stability of EVs for cancer-specific detection capabilities and accessibility^[66-68]^.

For instance, studies have identified high levels of EV markers such as CD63 and caveolin-1 in metastatic melanoma plasma EVs^[69]^. In the case of HNSCC, Theodoraki *et al.* demonstrated a correlation between high plasma EV PD-L1 levels and disease progression, predicting poor outcomes in a cohort of 40 HNSCC patients^[70]^. Panvongsa *et al.* identified miR-491-5p as a diagnostic and prognostic marker from plasma EVs in a dynamic model distinguishing HNSCC patients with locally advanced cancer from healthy controls^[71]^. Tables 3 and 4 summarize studies including pretreatment of blood EVs and further elaborate on the sampling process techniques, isolation of EVs, detection methods, and report potential EV biomarkers.

**Table 3:** Summary of blood EVs pretreatment in OSCC

**Type of collection tube**	**Volume of sample**	**Sample** **filtering**	**Sample processing**	**Sample volume and temperature for storage after pretreatment**	**Reference**
Venous blood tube	1 mL	No	0.5-mL aliquots of serum were loaded onto Sepharose 2B column (size exclusion chromatography), equilibrated with PBS. 1 mL of eluted fractions was further centrifuged at 10,500 x *g* for 1 h at 4 ℃ to obtain the EV pellet	1 mL/ --	Kim *et al.,* 2005^[17]^
--	--	No	Samples were isolated using membrane affinity column to isolate EVs. Plasma was mixed with binding buffer in a 1:1 ratio and loaded onto the membrane affinity column. After centrifugation and getting the flow-through, samples were washed with various buffers and EVs were eluted into a clean tube using 400 µL of buffer	--/--	Peng *et al.,* 2018^[27]^
Vacuum blood collection tubes (Insepack II)	1 mL	No	1 mL of sample was diluted 1:2 with PBS and centrifuged at 10,000 x *g* for 10 min at 4 ℃ Subsequently, samples were centrifuged at 100,000 x *g* for 70 min twice to obtain EV pellets	--/--	Nakamichi *et al.,* 2021^[24]^
--	500 µL	No	500 µL of serum was resuspended in 500 µL of PBS and processed using precipitation solution following the manufacturer’s instructions	--/--	Li *et al.,* 2019^[28]^
EDTA Vacutainer® blood collection tubes	2 mL for non-cancer controls. 1.6 mL for non-nodal oral tongue cancer patients. 1.2 mL for nodal oral tongue cancer patients	No	Samples were centrifugated at 1,600 x g for 20 min, 16,000 x *g* for 10 min, and 10,000 x *g* for 10 min 900 µL of pooled plasma was diluted with 1.1 mL of filtered PBS and then the sample isolated by size exclusion chromatography (Izon) Fractions were pooled and concentrated using Amicon filtration units	100-200 µL /-20 ℃	Qu *et al*., 2021^[29]^
	2 mL aliquots	0.22 µm	Fresh samples were centrifuged at 1,000 x *g* for 10 min. Thawed samples were centrifuged at 2,000 x *g* for 10 min. Subsequently, the samples were centrifuged for 10,000 x *g* at 4 ℃ for 30 min 1 mL of precleared samples were individually loaded onto size exclusion chromatography column (Sepharose 2B)	2 mL/-80 ℃	Ludwig *et al.,* 2020^[30]^

EVs: extracellular vesicles; OSCC: oral squamous cell carcinoma.

**Table 4:** Blood EVs included in OSCC

**Isolation Method(s)** **(U)** **(P)** **(S)** **(O)**	**Validation**	**EV Marker(s)**	**Detection Method(s)**	**Potential Biomarkers in EVs**	**Reference**
U, S	^a^^,^^b^Western blotting	Nil	Flow cytometry	FasL+	Kim *et al.*, 2005^[17]^
P	^a^ ^c^	CD9, CD63	^d^miRNA array	miR-34a-5p, miR-130b, miR29c-3p, miR-144-3p	Peng *et* *al.*, 2018^[27]^
U	^a^^,^^a^with immunogold labeling ^c^Western Blotting	CD9, CD81, CD63,	^e^	Alix	Nakamichi *et al.,* 2021^[24]^
P	^a^Flow NanoAnalyzer, Western Blotting	CD9, CD63, HSP70	^d^, ^e^, ^f^	PF4V1, F13A1, ApoA1	Li *et al.*, 2019^[28]^
S, O	^a^^,^^c^Western Blotting	CD9, Calnexin, TSG101	^f^	platelet factor 4 variant, tubulin beta-4A chain, histone H2B type 2-E collagen alpha-1(I)	Qu *et al.,* 2021^[29]^
S	^a^Western Blotting	TSG101	^g^	adenosine, inosine, hypoxanthine, xanthine	Ludwig *et* *al.*, 2020^[30]^

EVs: extracellular vesicles; OSCC: oral squamous cell carcinoma; U: ultracentrifugation; P: precipitation; S: size exclusion chromatography; O: Filtration / tunable resistive pulse sensing (qNano); ^a^TEM: Transmission Electron Microscopy; ^b^ITM: immunoelectron transmission microscopy; ^c^NTA: nanoparticle tracking analysis; ^d^qPCR: quantitative polymerase chain reaction; ^e^ELISA: enzyme-linked immunosorbent assay; ^f^LC-MS/MS: liquid chromatography-tandem mass spectrometry; ^g^UPLC/MS: ultraperformance liquid chromatography/ tandem mass spectrometry.

#### Plasma EVs as potential biomarkers in OSCC

In the outlined six studies on plasma EVs as potential biomarkers for OSCC [Table 3], the majority (50%) utilized size exclusion chromatography for purification, compared to membrane affinity binding, ultracentrifugation, and precipitation kits, each used in one study (16.67% each). Protein biomarkers were most frequently reported (66.66%) among various omics compared, followed by microRNAs and metabolites, each reported in one study (16.67% each). These findings highlight the potential of plasma EVs as screening biomarkers for clinical applications. Transmembrane proteins are important in the immune response^[72]^. Kim *et al.* collected plasma EVs from twenty-seven OSCC patients and twenty healthy patients, using size exclusion chromatography and ultracentrifugation. They initially identified that sera EVs from OSCC patients contained Fas ligand (FasL)^[17]^, a transmembrane glycoprotein involved in immune cell apoptosis^[72]^. Western blotting and immune-TEM further validated the presence of FasL displayed on EVs. Functionally, Fas-L in plasma EV samples induced caspase-3 activation, and the apoptotic pathway was inhibited by Z-VAD-FMK, a typical pan-caspase inhibitor, confirming the role of caspase-3 in intrinsic apoptotic signaling. Blocking EV FasL with antibody ZB4 also partially inhibited EV biologic activity. Moreover, comparing the levels of FasL expression in patient plasma EVs with their clinicopathologic characteristics, revealed that patients with late-stage cancer had higher FasL expression, which benefits as potential OSCC biomarkers. The expression of certain miRNAs in plasma-derived EVs is observed in cancer progression and is associated with patient outcomes, at least in gastric cancer^[73]^. The dysregulation of miRNAs is a hallmark of cancer, contributing to cancer progression, invasion, and metastasis^[74]^. One study by Peng *et al.* identified miRNA expression in OLP plasma-derived EVs. The study evaluated plasma from 19 OLP patients and 11 age-sex-matched healthy individuals. Plasma EVs were isolated using precipitation kits. The data showed significant upregulation of miR-34a-5p and miR-130b in EVs, while miR-29c-3p and miR-144-3p were downregulated compared to healthy controls. Bioinformatic analysis identified the PI3K/ Akt signaling pathway to be involved in OLP progression. Clinical evaluation using the reticular, atrophic, and erosive lesion (RAE) scoring system correlated plasma EV miR-34a-5p with the clinical diagnosis of OLP^[27]^.

Using quantitative proteomic analysis, serum EVs from 30 healthy individuals and 60 OSCC patients with or without lymph node metastasis were assessed^[28]^. EVs were isolated from serum using precipitation kits. Proteomic analysis revealed thirty-seven differentially expressed proteins, including 20 upregulated proteins and 17 downregulated proteins in OSCC patients without lymph node metastasis, and twenty-eight differentially expressed proteins with 15 upregulated proteins and 13 downregulated proteins in those with lymph node metastasis. The data showed that EV proteins PF4V1 was negatively correlated with lymph node metastasis, F13A1 was correlated with positive lymph node metastasis, while ApoA1 was associated with patients who had a history of smoking and drinking. Comparing protein profiles with diagnostic models, receiver operating characteristic (ROC) curve analysis validated F13A1, ApoA1, and a combination of F13A1 and ApoA1 EV biomarkers that aid in predicting metastases in OSCC.

Recent papers reported that plasma EVs are preferred in research studies, instead of serum EVs, as the membranous microvesicles inside the serum test tubes are reported to perform a type of self-secretion during clot formation, which is not cancer-specific. This blood tube collecting method may not be a good source for exploring biomarker studies^[75]^, although the techniques described often lacked detail. To get a better source of plasma EVs for determining the profiles of OSCC patients, our colleagues demonstrated a complete fourteen cases of proteomes profile of plasma sample EVs, by comparing non-cancer controls, OSCC non-nodal and nodal involvement^[29]^. Plasma EVs were isolated through size exclusion chromatography and filtration. Particle quantification by nanoparticle tracking analysis (NTA) showed a higher EV concentration from OSCC patients with lymph node metastasis than the non-cancer controls. In addition, two-dimensional high-performance liquid chromatography and subsequent tandem mass spectrometry analysis revealed 43 unique, dysregulated proteins in OSCC plasma EVs compared to non-cancer controls. Among the 43 plasma EV protein candidates, four proteins, platelet factor 4 variant, tubulin beta-4A chain, histone H2B type 2-E, and collagen alpha-1(I), were suggested as potential biomarkers in OSCC, due to their informative significance.

Metabolic cargo in EVs shows great promise to act as biomarkers in cancer; however, EV metabolites are still in their infancy in OSCC^[76]^. Recently, Ludwig *et al.* noted relatively elevated levels of genes encoding purine synthesis pathway members in HNSCC tumors vs adjacent normal tissues. This led to a search for reported purine metabolites in patient plasma EVs, which may be involved in tumor immune escape^[30]^. EVs were isolated from the blood of 26 OSCC patients and five healthy individuals using size exclusion chromatography (SEC), and were subsequently analyzed by ultraperformance liquid chromatography (UPLC). The analysis revealed significantly higher levels of purine metabolites in the plasma EVs of HNSCC patients, including some with OSCC. Metabolites include adenosine, inosine, hypoxanthine, and xanthine, particularly in cases of early-stage disease. The results showed metabolites as cargo in EVs can act as biomarkers in OSCC. Tables 3 and 4 summarize blood EVs pretreatment [Table 3] and the methods utilized for EV isolation and detection, as well as potential OSCC biomarkers [Table 4].

### Lymphatic fluid EVs

Lymph node metastasis is a common occurrence in OSCC^[77]^. Standard treatment includes surgical resection of lymph nodes followed by adjuvant radiation with or without chemotherapy^[78]^. Post-surgery, lymphatic fluid is drained to prevent wound seroma collection^[79,80]^.

Previous studies have reported the collection of post-surgical lymphatic fluids in carcinomas such as breast cancer, cutaneous melanoma, and thyroid cancer^[81-85]^. These lymphatic exudates were found to be rich in EV proteins^[86,87]^. Ekstrom *et al.* reported that lymphatic fluid-derived EVs from breast cancer patients were enriched with cancer-related EV markers CD29, CD44, and CD146^[86]^. Garcia-Silva *et al.* assessed the efficacy of EVs derived from lymphatic fluid compared to those from plasma in melanoma patients for biomarker detection. NTA revealed a higher number of EVs derived from lymphatic fluid compared to plasma, EVs from lymphatic fluid exhibited more polydisperse sizes than those from plasma, whereas mass spectrometry detected TRP-2 EV protein in both plasma and lymphatic fluid EVs. This suggests that lymphatic fluid EVs are potential markers in cancers.

#### Lymphatic fluid EVs as potential markers in OSCC

As a rich source of EVs, the lymphatic fluid serves as a great alternative for liquid biopsy in OSCC^[18]^. Our group recently assessed the biomarker potential of lymphatic fluid EVs. Lymphatic fluid was postoperatively collected from OSCC patients and plasma was collected from OSCC patients with or without lymph node metastasis, as well as from healthy individuals. Our data showed specific lymphatic fluid EV marker CD24 was present in OSCC patients with lymph node metastasis, suggesting that lymphatic fluid EVs may contain potential biomarkers in OSCC.

## SUMMARY AND PERSPECTIVES

**Figure 4:**
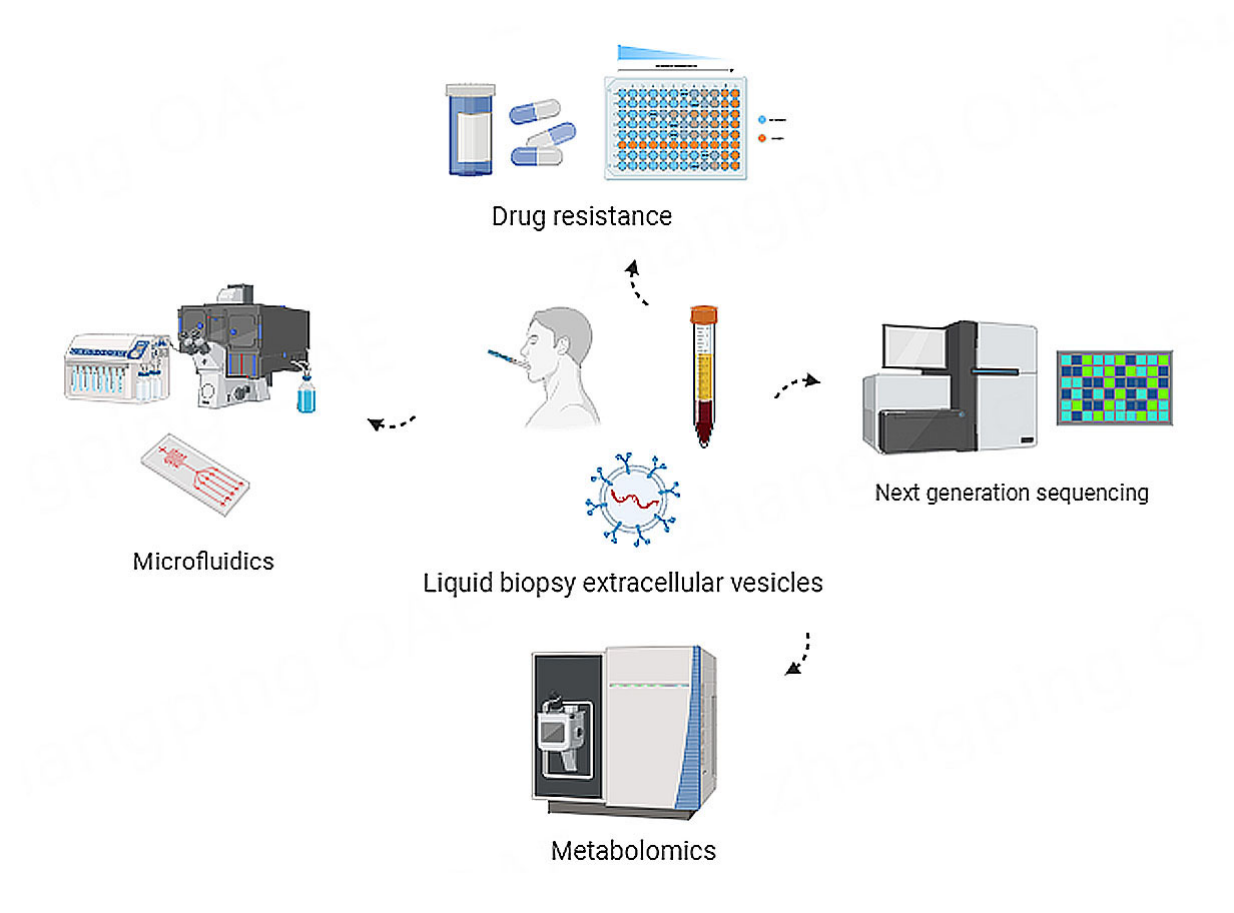
Overview of research gaps of liquid biopsy EVs in OSCC. There are four key areas where research on liquid biopsy EVs in OSCC is lacking, including drug resistance, microfluidics, metabolomics and next-generation sequencing. EVs: extracellular vesicles; OSCC: oral squamous cell carcinoma.

This review suggests that liquid biopsy enables the non-invasive capture and analysis of EVs. We highlight their potential as an alternative diagnostic tool to traditional tissue biopsies. However, the clinical development of liquid biopsies for EV detection is still limited. We aim to elaborate on the research gaps and future perspectives to aid in future diagnostic and prognostic utility [Figure 4].

### Lack of multi-omics studies with liquid biopsy EVs in OSCC

Multi-omics analysis provides an in-depth look into multiple levels of cells. Studies have employed genomics, transcriptomics, proteomics, metabolomics, and radiomics for cancer prognostic predictions^[88,89]^. However, multi-omics on EVs has not been thoroughly assessed, as most of the studies only focus on transcriptional and translational analysis.

Next-generation sequencing of HNSCC and OSCC for somatic mutations has identified promising cancer markers in liquid biopsies, which are crucial for biomarker screening^[90,91]^. A study used HNSCC tumor tissues and paired whole-blood samples to detect tumor-specific somatic mutations through whole exome and targeted sequencing, while saliva samples were analyzed using targeted deep sequencing for cell-free DNA detection^[91]^. A comparison of tumor tissue-specific somatic mutations with germline mutations from whole blood revealed frequent mutations in TP53, CASP8, AJUBA, CDKN2A, and NOTCH1. Surprisingly, whole genome sequencing did not detect TP53 mutations in tumor tissue, but targeted deep sequencing detected TP53 mutations in matched saliva cell-free DNA. These findings suggest that liquid biopsy biomarkers serve as valuable indicators for OSCC in clinical management.

In a larger cohort study of 121 OSCC patients, next-generation sequencing was performed on primary tumor tissue and matched saliva to assess single nucleotide variants and DNA content^[90]^. The most frequent mutations detected in both primary tumors and saliva were TP53, CDKN2A, FAT1, CASP8, and NOTCH1, while the addition of PIK3CA and HRAS to the panel increased the frequency variants in a tumor-specific mutation population-based screening. Hence, liquid biopsies of tumor-derived DNA may provide a rapid, sensitive, cost-efficient, and non-invasive method for early detection in high-risk OSCC populations.

Due to the proximity to primary tumors, adjacent tissues in OSCC patients may also undergo malignant transformation. Shanmugam *et al.* reported that histologically abnormal tissues adjacent to tumors often exhibit multiple gene mutations, chromosomal instability, loss of heterozygosity, and microsatellite alternations^[90]^. Peripheral blood, saliva, and adjacent tumor mucosa tissues from 27 OSCC patients were analyzed using next-generation sequencing. Liquid biopsy data from saliva (circulating) tumor DNA was compared with adjacent tumor mucosa samples. Targeted capture sequencing revealed that TP53 and FAT1 gene mutations were highly expressed in both tissues and liquid biopsies, demonstrating the potential for accurate detection of gene mutations in liquid biopsies that could inform clinical practice.

Although studies evaluating metabolomics and radiomics of OSCC EV samples are limited, existing research has primarily focused on various detection methods of liquid biopsy EVs using transcriptomics and proteomics platforms. For instance, studies on salivary EVs have utilized miRNA arrays for detection, confirmed through qPCR or small RNA sequencing^[15,21,92]^. Proteomics analyses have detected target EV proteins using LC-MS/MS and/or ELISA^[22,24]^. Similarly, plasma EVs have been examined in studies profiling miRNA and proteomics^[27-29,92]^.

### Lack of studies in OSCC reporting the advanced technology to isolate liquid biopsy EVs by Microfluidics

The prospects of microfluidics-based EV isolation are promising; however, its utilization in OSCC research has not been implemented. Various microfluidic systems for detecting EVs have been developed, such as acoustic nanofiltration, deterministic lateral displacement, filtration, immunoaffinity, nanowire trapping and viscoelastic flow, as they offer the advantage of requiring smaller volumes for EV collection^[77]^. They also enable the separation of EVs at the nanoscale in minimal volumes, simplifying the isolation process from complex biofluids^[78]^. However, microfluidic techniques face challenges in effectively separating impurities, particularly protein complexes and aggregates that can overlap with small EVs based on size and density^[79]^. Therefore, stringent purity criteria are essential when using microfluidics to isolate EVs from biofluids. In OSCC, there is a notable absence of studies reporting the isolation of EVs using microfluidics. As microfluidic-based technologies gain traction in other areas of cancer research, investigating this detection method in OSCC becomes imperative.

### Lack of studies on liquid biopsy EVs and drug resistance in OSCC

Cisplatin is a cost-effective chemotherapeutic agent for OSCC, but some patients inevitably experience tumor progression or recurrence, often accompanied by increased tumor invasiveness. Clinical studies have reported recurrence rates ranging from 18% to 76%, with most recurrences observed within 2 years post-treatment^[93]^. Cancer-derived EVs act as carriers that facilitate communication between tumor cells, promoting angiogenesis and metastasis. While several studies have described EV-mediated chemoresistance in OSCC cells, little research has focused on liquid biopsy EVs. Among the genetic cargo, miRNAs in EV biomarker profiles appear to play a significant role in drug resistance in OSCC cells. For instance, EV miR-21 confers cisplatin resistance by targeting PTEN and PDCD4 in OSCC cells in vitro and in vivo^[93]^. Kalluri *et al.* demonstrated that EV miR-155 in cisplatin-resistant OSCC cells enhances cell migration and invasion. Moreover, EV miR-155 upregulates the expression of mesenchymal markers such as N-cadherin, β-catenin, Twist, and vimentin^[94]^. We anticipate that future studies on liquid biopsy EVs will elucidate their role in drug resistance mechanisms in OSCC.

### Liquid EVs as potential drug targets and future perspectives

While OSCC-derived EVs are primarily studied for biomarker detection, they also hold promise for therapeutic purposes. EVs exhibit low immunogenicity, possess inherent targeting abilities, and can be engineered for drug loading^[95]^. They can transport small molecules for cell absorption through circulation and participate in various biological responses^[96]^. EVs are proposed for treating diverse diseases through cargo loading. For example, clinical trials are underway to determine the maximum tolerated dose, toxicities, and disease impacts of iExosomes carrying silencing RNA to target oncogenic KRAS in patients with pancreatic cancer. The generation of these “iExosomes” has been described^[97]^. In ascites fluid from gastric cancer cells, EVs from MET-expressing cells can increase invasion and angiogenesis in both MET-amplified and non-amplified tumor models. Depletion of MET in cells by silencing RNAs also reduced MET in EVs from those cells, and delivery of such EVs reduced tumorigenic behavior in MET-expressing gastric cancer cell models^[98]^

In addition to genetically engineered EVs, EV-mimetic drug carriers have been proposed as ideal natural formulations for targeted drug delivery. For instance, oral delivery of bovine colostrum-derived EVs packed with paclitaxel (ExoPAC) increased the efficacy compared to conventional paclitaxel infusion in lung cancer cells^[99,100]^. As mentioned above, Mendt *et al.* developed clinical-grade healthy bone marrow-derived mesenchymal stem cell EVs following good manufacturing practice (GMP) standards. These EVs were loaded with siRNAs intended to target the Kras^G12D^ mutation in pancreatic cancer^[97]^. In OSCC, bovine milk-derived EVs embedded with a light-sensitive drug system enhanced anticancer activity against OSCC cell lines, HSC-3, SCC-9, and Cal-27. Due to their relative lack of immunogenicity, bovine milk-derived EVs hold promise as a cost-effective and non-toxic drug delivery vehicle. They are recommended as a potential therapeutic approach for OSCC and possibly other cancers^[101,102]^.

## CONCLUSION

In conclusion, EV-based liquid biopsies are increasingly important for the detection of OSCC. This review identified thirty-eight and fifteen potential biomarkers detected in saliva and plasma EVs, respectively. Among the potential biomarkers, EV proteins were the best-reported targets for the detection of oral cancer, accounting for 25 out of 38 targets (66%) in saliva and 11 out of 15 targets (73%) in plasma.. Although most studies traditionally focus on tissue and circulating DNA analysis, there is a substantial body of literature demonstrating that EVs can effectively preserve genetic materials for long-term storage. Given the common occurrence of lymph node metastases in OSCC, real-time collection of liquid biopsy EVs may offer an accurate method for identifying prognostic biomarkers and determining prognosis.

Although EV detection in OSCC is still in its infancy, future perspectives advocate for standardized detection protocols for EV-based liquid biopsies to enhance sample sensitivity and specificity. Improving detection accuracy will require larger cohort studies for sample collection and advanced omics analyses. Furthermore, for future clinical applications, studies should consider longitudinal collection periods from OSCC patients, spanning from diagnosis through surgical procedures to post-surgical radiotherapy and chemotherapy to obtain precise EV biomarkers for cancer diagnosis and prognosis.

Regarding translational/therapeutic implications, while the therapeutic use of EVs in OSCC treatment remains underdeveloped, scaling up drug development efforts could be feasible once potential EV therapeutic and prognostic markers for OSCC are identified for further translational exploration.

## DECLARATIONS

### Copyright

© The Author(s) 2024.
